# Stakeholders in support systems for self‐care for chronic illness: the gap between expectations and reality regarding their identity, roles and relationships

**DOI:** 10.1111/hex.12471

**Published:** 2016-05-27

**Authors:** María J. Pumar‐Méndez, Agurtzane Mujika, Elena Regaira, Ivaylo Vassilev, Mari Carmen Portillo, Christina Foss, Elka Todorova, Poli Roukova, Ingrid A. Ruud Knutsen, Manuel Serrano, Christos Lionis, Michel Wensing, Anne Rogers

**Affiliations:** ^1^ School of NursingUniversity of NavarraNavarraSpain; ^2^ Quality DepartmentClínica Universidad de NavarraNavarraSpain; ^3^ Faculty of Health SciencesNIHR Wessex CLAHRCUniversity of SouthamptonHampshireUK; ^4^ Institute of Health and SocietyUniversity of OsloOsloNorway; ^5^ Department of Economic SociologyUniversity of National and World EconomySofiaBulgaria; ^6^ Department of Economic and Social GeographyNIGGGBulgarian Academy of SciencesSofiaBulgaria; ^7^ Education, Health and Society FoundationMurciaSpain; ^8^ Clinic of Social and Family MedicineDepartment of Social MedicineUniversity of CreteHeraklionGreece; ^9^ Radboud University Medical CentreRadboud Institute for Health SciencesNijmegenThe Netherlands

**Keywords:** chronic illness, cross‐national research, patient‐centred care, self‐care, self‐management support, stakeholders

## Abstract

**Background and objective:**

The spread of self‐care holds the promise of containing chronic illness burden. Falling within the framework of a FP7 collaborative research project, this paper reports the views of key informants from six countries regarding who the main stakeholders are at different levels in the support system for self‐care for patients with chronic illness (SSSC) and how they accomplish their role and collaborate.

**Methods:**

90 Interviews with purposefully selected key informants from Bulgaria, Greece, the Netherlands, Norway, Spain and United Kingdom were conducted. Interviews involved government and local authorities, politicians, academics, health professionals and private sector representatives. Interviewers followed an expert opinion‐based guide. Analysis involved a cross‐country examination with thematic analysis and framework method techniques.

**Results:**

Key informants described the ideal SSSC as inclusive, interdependent and patient‐centred. The following main stakeholders in SSSC were identified: patients, governments, health‐care professionals, associations, private companies and the media. In the current SSSCs, collaboration among stakeholders within and across different levels was said to be lacking. Patients were seen as playing a passive subordinate role based on the following: their own attitudes; the paternalistic and medicalized attitudes of the health‐care professionals; their misrepresentation by patient associations; and their exposure to the damaging influences of media and industries.

**Conclusions:**

Making SSSC patient‐centred constitutes the greatest challenge for European authorities. Strategies must be revised for promoting patient participation. They should undergo changes so as to promote industry and media social responsibility and patient association advocacy capacity.

## Background

Fast‐moving social and demographic changes in recent years have imposed great challenges on health systems. A critical example of this is the enormous and rising prevalence of chronic illness which, according to the World Health Organisation (WHO), could account for 57 percentage of the global burden of disease by 2020.^1^ Because such a burden would be overwhelming and posing a threat to the sustainability of health systems, new strategies for tackling chronic illness are emerging. Among the latter is self‐care, which can be understood as ‘*the care taken by individuals towards their own health and wellbeing [that] comprises the actions they take to lead a healthy lifestyle; to meet their social, emotional and psychological needs; to care for their long‐term condition; and to prevent further illness or accidents’*.^2^

Evidence suggests that the main intermediate objective of self‐care, sustainable behaviour change, can be better attained through multilevel approaches (individual, community, organizational and systemic levels) that address processes involved in illness management at different systemic levels.^3^, ^4^ This is not surprising because findings from qualitative research have revealed that to engage in self‐care tasks, patients feel the need for different types of support, stemming from a variety of sources (e.g. Instrumental, psychosocial and relational support from health‐care professionals, relatives and peers).^5^ The corollary is that the development and deployment of self‐care strategies require the involvement and coordination of multiple stakeholders at different systemic levels.

Moreover, this has implications for the design of support systems for self‐care for patients with chronic illness (SSSCs), an endeavour that policymakers and governments across Europe have gradually incorporated into the broader agenda of public health, health promotion and patient‐centred care.^6^, ^7^ In particular, the main implication is that SSSC should adopt a social–ecological approach that supports patients and their capacity for self‐care by addressing not only individual factors but also environmental influences spanning macro‐, meso‐ and micro‐contextual levels. Indeed, environmental influences such as governance arrangements within welfare and health‐care systems (macro‐level), services provided by voluntary and community organizations (meso‐level) and patient domestic and employment context characteristics (micro‐level) have been identified as influencing self‐care support.^6^, ^8^

The operationalization of this social–ecological SSSC is challenging. While research abounds in terms of how support for self‐care is influenced by individual factors, understanding the impact of environmental influences remains scarce.^9^ This makes it difficult to establish which environmental aspects should be prioritized in the design of SSSC and who could and should be involved and held responsible for their management.

In summary, it is necessary to broaden our understanding regarding how support for self‐care is influenced by environmental factors in order to facilitate the design of SSSC, thus allowing for the implementation of informed initiatives relevant in the everyday life of individuals. To accomplish this general aim across selective settings in Europe, a project funded under the EU's 7th Framework Programme, entitled EU‐WISE (Self‐Care Support for People with Long Term Conditions, Diabetes and Heart Disease: A Whole System Approach)^10^ included an exploration of the influence of the broader socio‐economic and policy environment on the capacity of self‐management. More specifically, this investigation included the identification and examination of views expressed by key informants in relation to (1) emerging policies and practices regarding type 2 diabetes and self‐care; (2) impact of macro‐ and meso‐level influences on the SSSC for type 2 diabetes; and (3) roles, division of labour and relationships of stakeholders on the micro‐, meso‐ and macro‐level of the SSSC for patients with chronic illness and type 2 diabetes.

Most relevant findings related to the views expressed by key informants on policy practices and meso‐level influences on type 2 diabetes and self‐care have been reported elsewhere.^9^, ^11^ This paper focuses on reporting the findings related to the views of key informants from six European countries regarding who the main stakeholders are at different levels in the SSSC and how they should ideally participate and interact among one another. Key informants' perspectives on the actual levels of coordination and collaboration between these stakeholders are also examined.

## Methods

The EU‐WISE exploration of the roles, division of labour and relationships of stakeholders in the SSSC for patients with chronic illness and type 2 diabetes involved interviews with key informants from a range of socio‐economic, institutional and health‐care contexts that could influence the organization of and experiences with SSSC. These contexts were the EU‐WISE project partner countries: Bulgaria (BG), Greece (GR), the Netherlands (NL), Norway (NO), Spain (ES) and United Kingdom (UK). Each partner country obtained ethical approval for the project from their pertinent Ethics Committee. A pan‐European approach to study the issue was preferred as it can provide more robust insight into context‐dependent phenomena than single studies and it accelerates the generation, accumulation and transfer of knowledge across countries. Furthermore, it offers the opportunity to identify a basic set of networking structures and practices that suggest suitability for different contexts, and thus, can enrich the development of supranational strategies and policies directed at strengthening SSSCs.

The selection of key informants was purposeful and aimed at maximizing variation and expert sampling. A special effort was made to include a wide variety of participants from different fields who had first‐hand inside knowledge regarding policy, structures and practices related to self‐care support for chronic illness and type 2 diabetes. These participants included government representatives and local authorities, as well as politicians, academics, health professionals (i.e. general practitioners, specialist physicians, nurses, pharmacists and dieticians) and representatives of the private sector (i.e. drug, technology, food and insurance companies). Patients were not included because although they could offer a different perspective on the issues under study, their opinions would not be based on expertise in terms of informing, shaping and spreading the uptake of practices and policies related to health programmes.

Potential key informants were identified through personal knowledge of project team members, snowballing techniques and examination of policy statements and organizational websites in each partner country. Once identified, potential key informants were approached via telephone calls or emails and given a brief explanation of the project and interview topics. Further information and a consent form were emailed through a second contact, after which approval to participate was obtained and interviews were scheduled.

Table 1 presents details on the backgrounds of the 90 key informants interviewed (15 per partner country).

**Table 1:** Key informants' background^a^

	Health professional			Policymaker/politician	Academic	Industry representatives (Drug/Tech)/health facilities managers
	General practitioner/specialist	Nurse	Other (pharmacist, dietician)			
Bulgaria	11	1	3	5	5	5
Greece	6	2	7	3	3	3
The Netherlands	2	2	11	6	3	1
Norway	5	4	6	7	2	2
Spain	5	3	7	6	7	3
UK	6	1	8	3	9	3

a 15 key informants per country who, in many cases, could be described under different categories.

Interviews were conducted face to face or via telephone by project team members or thoroughly trained interviewers who followed an interview guide based on expert group discussions. As summarized in Table 2, the latter was adapted to each partner country and included questions reflecting the main interview topics. Interviews lasted between 30 and 90 min.

**Table 2:** Interview guide (questions adapted to each partner country)

What are the key changes, policies, innovations in self‐care support and diabetes type 2 over the last 10 years? Why have these been the most important ones? What changes have these led to? Why do you think policy has changed in the way that it has? Who are the most important stakeholders in this area? How have they influenced the agenda around self‐care support? What is the role of drug companies nationally/internationally? Do you have a view of current policy around the role of drug companies or how they influence the agenda in this area? What is the role of telecare companies? What is the involvement of other private companies in self‐care support? How is the broader health‐care system organized? What are the public attitudes to self‐care support and diabetes type 2? What are the media constructions of the epidemic of diabetes type 2 and who is at risk?

Interviews were audio‐recorded and transcribed verbatim into Bulgarian, Greek, Dutch, Norwegian, Spanish and English. Some of the Dutch interviews were not audio‐recorded. The latter were analysed on the basis of detailed summaries typed immediately after each interview. Anonymity of key informants' contributions was maintained in the presentation of the data.

The analysis of the transcripts involved a three‐stage cross‐country examination guided by thematic analyses and techniques from the framework method.^12^, ^13^. Each partner country provided a preliminary analysis of a set of interviews that were examined together so that consistent themes and topics could be identified across countries, leading to the emergence of a common thematic framework. Following the reading of the transcripts and field notes, each partner country undertook a thematic and textual intracountry analysis that led to the identification of recurring themes and subthemes. Selected quotes illustrative of these themes were translated into English to allow for discussion among partner countries in two comparative cross‐cultural data analysis clinics and for supplementary discussions with individual partners. The initial coding of each country's data set was subjected to an adapted comparative method to identify convergent and divergent themes across topics. Project team members from each country accounted for cross‐cultural differences in the data sets while working towards shared meanings to reach a consensus on the meanings of key topics.

## Findings

Two main themes emerged in relation to the roles, division of labour and relationships of stakeholders in the SSSC for patients with chronic illness and type 2 diabetes. The first theme, ‘Identity of stakeholders in the SSSC’, reflects the views expressed by key informants regarding who the main stakeholders are at different levels in the SSSC and how they should ideally operate and interact. The second theme, ‘Attitudes and collaboration of stakeholders in the SSSC’, reflects the perceptions of key informants concerning how these stakeholders actually recognize the roles assigned to them and how they are currently acting, coordinating and collaborating to support self‐care. Therefore, the second theme abandons the descriptions of the key informants' expectations on how the SSSC should operate to focus on their perceptions of the actual state of affairs.

### Identity of stakeholders in the SSSC

**Figure 1:**
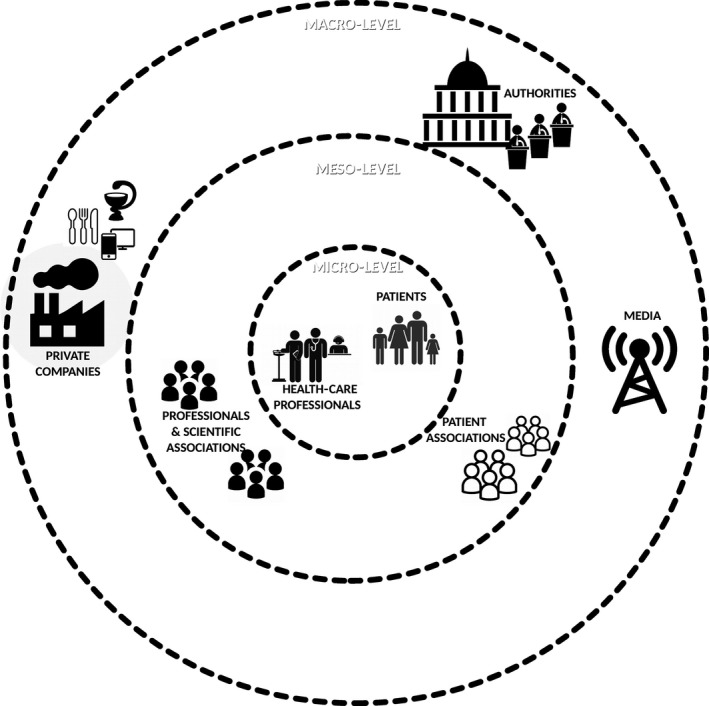
Stakeholders at different levels in the SSSC.

According to the key informants, if new chronic illness and type 2 diabetes strategies (and subsequently self‐care) are to be promoted, multiple stakeholders should intervene in a complementary and coordinated manner. Among these stakeholders, key informants cited patients, governments, health‐care professionals, professional, scientific and patient associations, private companies (such as drug, technology and food companies) and the media. As presented in Fig. 1, these different stakeholders belong to and operate at different SSSC levels, including the micro‐, meso‐ and macro‐levels.

**Figure 2:**
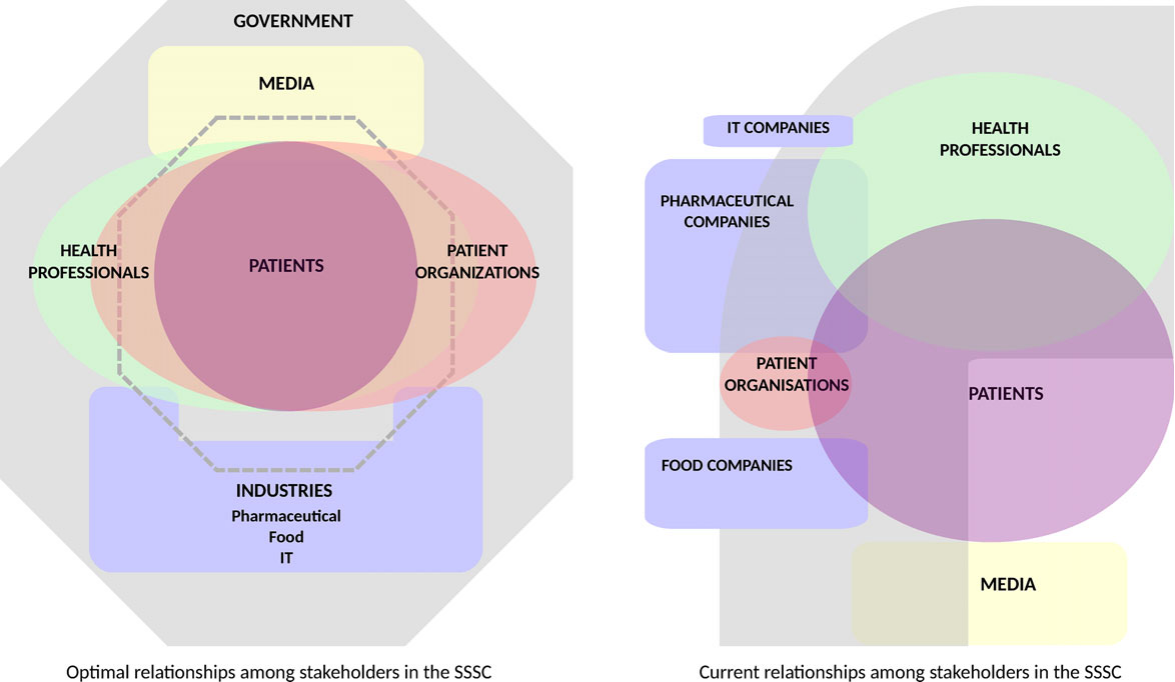
Optimal and current relationships among stakeholders in the SSSC as described by key informants.

Key informants described different functions for each of these stakeholders that, far from being hermetic, are complementary and interdependent and thus prescriptive of a particular pattern of interactions within the SSSC. The optimal relationships among stakeholders in the SSSC for patients with chronic illness and type 2 diabetes as described by key informants are presented in the left diagram of Fig. 2.

As illustrated in the left diagram of Fig. 2, key informants from the six countries were consistent in stating that the SSSC should be patient‐centred. In this arrangement, all efforts and resources for self‐care are organized around patients who, given their central position, become empowered to establish active and direct relationships with health professionals and patient associations. These relationships are expected to give patients a means by which to make an impact not only on their own care but also on general standards of care, health services designs and health policies. Ultimately, key informants expressed that patients' empowerment and participation, aside from being desirable to themselves, are necessary for meeting the demand for increasing the responsibility of patients in their own health management and decision making. While key informants from the UK and ES linked this demand with the need to contain burdens on the health‐care sector, Norwegian key informants linked it with a process of increased democracy.

From the key informants' perspectives, in the ideal SSSC, health‐care professionals should serve as some of the closest partners of the patients at the micro‐level (see left diagram of Fig. 2). Health‐care professionals are expected to focus on helping patients increase their autonomy to the maximum extent possible, thus allowing an effective implementation of self‐care.

Patient associations were described as the most important instruments for patients for influencing the health policies and practices of health professionals. They should maintain a close relationship with patients, advocating for patient aspirations and needs in terms of care and self‐care, and at the same time, they need to maintain ties and collaborate with health‐care professionals, professional associations and health‐care planners.

According to key informants, the input of patient associations and professional associations should help authorities at different levels to fulfil their roles in developing and maintaining the SSSC. These roles include the development and enforcement of self‐care, chronic illness and type 2 diabetes policies; the organization of health systems; the allocation and distribution of resources; and regulating and overseeing the environment wherein the SSSC is framed. This includes the regulation of industry (especially the pharmaceutical and food industries) and the media to protect the public from unhealthy environmental influences.

Key informants charged industry and the media with the duty of collaborating with health professionals and organizations so as to share accurate information about chronic illness and self‐care with the public, break myths associated with chronic illness and self‐care, and increase health literacy. More importantly, key informants highlighted the need for industry and the media to self‐regulate, fulfilling their social responsibility of avoiding environmental impacts that endanger public health.

In addition to being consistent about how the SSSC should be characterized as patient‐centred and inclusive, key informants also agreed that this idealized model is far from the current reality that is depicted by the right diagram in Fig. 2.

### Attitudes and collaboration of stakeholders in the SSSC

Within the theme regarding the current roles of the stakeholders, coordination and collaboration, three subthemes emerged highlighting the perceived deviations from the ideal model of SSSC, as observed by the key informants: (1) distortions in micro‐level stakeholder attitudes and relationships, (2) distortions in meso‐level stakeholder attitudes and relationships with stakeholders at the micro‐level and (3) distortions in macro‐level stakeholder attitudes and relationships with stakeholders at the micro‐level.

#### Distortions in micro‐level stakeholder attitudes and relationships

As presented in the right diagram of Fig. 2, key informants were in agreement that the central role of patients in the SSSC that had been urged had not been developed or put into practice. Instead, patients were seen as playing a passive and subordinate role due to influence from the media and the private sector and considered to be under the dominance of paternalistic health‐care professionals.

According to the key informants, the subordinate relationship with health‐care professionals was accepted and even promoted by large sectors of patients. For example, key informants from GR and ES stated that elderly people are used to having a paternalistic relationship with health‐care professionals and thus may not have the cultural inclination, experience, skills or health literacy to participate in decision‐making processes that affect their health and self‐care. Key informants from the UK noted that younger patients were also having problems taking on a proactive role in self‐care due to a lack of basic skills required to undertake simple self‐care tasks such as cooking. This may result in an increase in patient preferences for medicalized care that relies on expert instructions and control.

For type I diabetes things are different because the patients are young. In type II (…) it's extremely difficult to persuade someone that their health does not solely rely on the doctor's decision on the units of insulin they take in the doctor's office, but that they too have to do something. To a large extent it shifts the doctor‐patient relationship from how it had been previously established (GR1; Epidemiologist and internist)

People prefer more a paternalistic doctor that tells them what they have to take and what they have to eat (…) this demands from them less suffering and anxiety than having to learn to manage things for themselves, and it requires less effort on their part (…) I think this is a problem created by the doctor‐patient culture in this country, where people are used to that ‘Mr Doctor says I have to take these pills and I take them, and I don't want to know’ (…) it also depends on the type of population: now there are younger people who tend more to seek information themselves, but the average diabetic patient who finds themselves in this area of Endocrinology is over 70, and has had diabetes for 30 years now, and has always done whatever he was told to do, so it's hard for them to make their own decisions (ES6; Endocrinologist and academic)

There's also a lot of concerns about how as a nation, I'm not just talking Scotland here, I'm talking UK, is for how many people no longer even have very basic cooking skills and actually think that being able to cook means that you can do things like put something in a microwave (UK7; Academic)

Nevertheless, key informants explained that the subordinate position of patients is also perpetuated by health‐care professionals. Key informants stated that while health‐care professionals' attitudes towards promoting patient autonomy and self‐management are noticeably improving, their practice is still dominated by paternalism. This was mainly attributed to the overexposure of health‐care professionals to medicalized paradigms and to existing practical barriers for implementing patient‐centred approaches, such as insufficient time, training, incentives, autonomy and multiprofessional human resources.

The way doctors behave is paternalistic and condescending and preserves their status quo (…) It is necessary to change the consciousness of patients, the training of doctors, to alter the behavioral model of medical personnel and it is important to start with the education at the medical universities (BG10; Academic)

Doctors are so much under time pressure to see so many patients that writing a prescription is a lot easier than taking that extra 5 min to find them the proper service (UK13; Drug company representative)

The satisfaction felt by the patients due to the rendering of services which they would not otherwise have, is the only incentive that I see (…) In England recently, 8 years ago, financial and personal incentives regarding quality of life were given (…) and after 5 years there was some improvement in health indicators (GR7; General practitioner)

You don't have an agent for the implementation of those recommendations, you have an agent for the implementation of all the drug recommendations – they're called doctors (…) Where are all these behaviour change experts? (UK9; Social policy campaigner and academic)

More encouragingly, key informants from the UK and ES observed that nurses seem more inclined to participate in the development of less patronizing and more egalitarian relationships with patients and thus to support and pioneer the implementation of new self‐care strategies. Nurses' professional ethos and the opportunity that the spread of self‐care presents for the expansion of their professional role were highlighted as the reasons for nurses to take such a favourable stance.

#### Distortions in meso‐level stakeholder attitudes and relationships with stakeholders at the micro‐level

Key informants stated that patients are disempowered and relegated to a minor passive role in the SSSC, not only by their own attitude and their health‐care professionals' paternalistic and medicalized attitudes but also because they are misrepresented in patient associations. As presented in the right diagram of Fig. 2, in most countries (especially BG and ES), the patient association sector is small, fragmented and immature. Their own needs for development and survival lead them to focus on delivering services to patients rather than on advocating for them.

Look how many patients' organizations there are and each one lobbies for its own interests, but they are not interested in educating the patients and defending their rights (BG10; Academic)

They always saw themselves as advisory and then (…) the only way to grow was to deliver services (…) you've now got a not for profit organisation that has spun off to deliver self‐management and indeed commercial organisations who now say we can do it better than you (UK9; Social policy campaigner and academic)

In addition, their financial needs lead patient associations to strive for partnerships with stakeholders, such as professional and consumer associations and private companies. This has given rise to a shift in the agendas of patient associations from patient‐centred to one driven by the interests of industry and professionals.

It isn't the patients the ones who represent themselves, but the consumers. Traditionally, the perspectives of consumers and patients are radically different, consumers are generally supporting the government stance at all times, and the patients, obviously, are more aggressive in demanding what they consider they need (…) Nowadays, the patients don't have a great influence on diabetes or on any other illness (ES4; Drug company representative)

They're very (…) one sided because it seems like they work very closely with medical professionals so everything they worked on was related to health care professionals so there wasn't much going on in terms of (…) health psychology or other areas where there is research being done (UK8; Academic and policy advisor)

In the UK, where the patient association sector appears to be more advanced, key informants noted that truly patient‐driven associations tend to be single‐issue campaigns without the ability and interest in promoting and seeing the bigger picture, and as a result, they are disregarded and marginalized.

There is this other charity (…) which was set up to campaign against human insulin which they believed caused cancer and is a true patient organisation (…) they're not quite as mad. It's a very, very small group and they're marginalised as being nutty. I think they probably are the foundation for a proper patient group but they're minor, minor, tiny, tiny, considered nutty (UK4; Academic and policy advisor)

The corollary of the failure of patient associations to fulfil their role of representing patients' interests and views to the authorities is that the influence patients have over health‐related policies was considered to be rather marginal.

#### Distortions in macro‐level stakeholder attitudes and relationships with stakeholders at the micro‐level

Remarkably, stakeholders that should be placed in the most distant position from stakeholders at the micro‐level were identified by key informants as the ones that find ways to establish direct contact with them (See right diagram of Fig. 2). For example, pharmaceutical companies were said to strive for establishing direct contact with both professionals and patients by means of financing the training and research of professionals or by getting involved in patient associations and educational activities. In most of the participant countries (UK, BG, ES, GR), this was said to occur despite existing regulations restricting these relationships.

There is a company which teaches patients how to take their insulin at home. Now, no company has been contracted with official bodies which provide such assistance (GR1; Epidemiologist and internist)

Most of the pharmaceutical companies take part together with the medical experts in training modules for patients, the most active participants being the companies producers of insulin (BG15; Internist)

Key informants representing pharmaceutical companies alleged that their activities with professionals and patients are only intended to contribute to the SSSC while adding value and differentiating their products in the marketplace. However, the rest of the key informants reiterated that pharmaceutical companies have ulterior motives for their formation of close relationships with professionals and patients. For example, by organizing training and educational activities, pharmaceutical companies have the opportunity to introduce informational biases that help reinforce the culture of medicalization most favourable to their organizational interests: maximizing medication consumption and sales.

There is now more of a tendency in some spheres to actually put people on type 2 diabetes with insulin a bit earlier on (…) obviously general practitioners base their clinical decisions on guidelines but to some extent the guidelines are informed by the evidence (…) and unfortunately the evidence base that is currently available is predominantly driven by the pharmaceutical companies (…) there are lot more agendas at play than we might want to think (UK7; Academic)

I just see their role very much as funding the kind of research that ensures that their products get on to patient prescriptions in order to maximize profit (UK7; Academic)

One Spanish interviewee explained that pharmaceutical companies, unlike IT or insurance companies, have not understood the importance and irreversible character of recent changes to health systems, and thus, they have committed a strategic error by failing to identify a new market niche.

In the last forty years the pharmaceutical industry has not cured hardly anything. What it has done is to turn loads of patients into chronic patients. The drugs that are now out there pull in the same direction (…) An interesting thing that may happen to them is that they are launching products that they are not going to be able to sell because the market is changing. They don't understand that their customer organisation is going to be very different from the organisation they used to sell to. A company should divert its attention to the payer who is in control (ES10; Academic and policymaker)

IT, telecare and telehealth companies were better regarded by key informants; they explained that their interests are more aligned with the philosophical underpinnings of chronic illness and self‐care strategies. Thus, despite their incipient development and marginal impact on the SSSC, telecare and telehealth companies were welcomed into the system with less suspicion. Nevertheless, some key informants highlighted that the potential for telecare and telehealth strategies to enhance the system of support for self‐care would be missed if their focus on the design and development of their products shifted from a patient‐focused approach to a health‐care professional‐focused approach. Indeed, telecare and telehealth products that are commercialized only as tools for the convenience of professionals and to save them time could perpetuate paternalism in the relationships between professionals and patients.

The mobile phone intervention has a lot of potential but you've got to be very careful how you organise it because the classic model is send your information to the healthcare professional and they'll tell you whether you have a problem. That's totally disempowering and that's a very common telehealth model currently practiced (…) which was designed to be for the convenience of the physicians (UK9; Social policy campaigner and academic)

Finally, food companies and the media were noted as the least compromised in the advancement of SSSC. According to key informants, food companies' sales strategies and false advertising practices (i.e. presenting their products as almost medicinal) and media sensationalism are harmful to people's eating behaviours and to the public's image of chronic illness and type 2 diabetes.

You go to X supermarket and there is the trick: there is something like a ‘healthy best‐offer’ but there is still the offer of unhealthy food that creates diabetics and obese people (ES10; Academic and policymaker)

The labelling here (…) is ludicrous. Low fat. What does low fat mean? One or 2 kilocalories below the full fat version but still 5 times higher than having a piece of fruit (UK9; Social policy campaigner and academic)

Some news coverage are little ‘bombs’ that go off, to do this that or the other (…) all undermine our work a lot, because misinforming is very dangerous. As for the Internet, they come to my office having already decided what their therapy should be. It's tiring and it leads to mistakes and it undermines the doctor‐patient relationship (GR9; General practitioner and internist)

Type 2 diabetes has had a focus in the media and it has been a biased focus upon overweight and perspectives – well one eats too much and doesn't move; that's why you have got diabetes type 2. It is obviously a stigmatising situation for those with diabetes type 2 (NO8; Nurse, professional association leader and policymaker)

The responsibility for the negative relationships between the industries, the media and the public was laid on the industries and media but also on the authorities. While private companies were accused of lacking social responsibility and refusing to self‐regulate, authorities were charged with failing to adequately regulate and oversee the environment of the SSSC.

Other industries have somewhat started to self‐regulate, but it is the agri‐food industry which needs to do something serious about sugar and salt (ES10; Academic and policymaker)

The media do not reflect on their educational role (…) Most of them told us: ‘This is not our work. Our work is not educating or training people. This is your work. Our work is just to inform about whatever happens thanks to our freedom of press’ (ES10; Academic and policymaker)

It is a problem that the government and the Ministry of Health do not control food and nutrition supplements advertisements (BG4; Patient association manager)

Enough pressure is not being put on producers and markets (…) you can see all sorts of conflicts of interest, you just have to look at the Olympics (…) we're trying to promote healthy activity and all our major sponsors are McDonalds and Coke (…) you allow advertisers to make those sort of false links in people's mind between healthy activity and McDonalds (UK10; Academic and policymaker).

## Discussion

This study exposes the desires of the key informants for both reconfiguration and the creation of new modes of functioning within the SSSC as well as their views on the current state of affairs. While the SSSCs are at different developmental stages in the participating countries, various issues could be observed that were common to all of them. First, key informants from the six partner countries described the ideal SSSC as inclusive (in that it demands the participation of multiple stakeholders at the micro‐, meso‐ and macro‐levels), interdependent and patient‐centred. This archetype, presented in more detail in the left diagram of Fig. 2, emphasizes the importance of a range of interactions between stakeholders at the micro‐ and meso‐levels in the SSSC as proposed in the chronic care model for chronic care improvement.^14^ This demonstrates the great impact that the chronic care model has had on European stances in the battle against chronic illness.

Furthermore, key informants from all of the participating countries described practical realities that are far from the ideal SSSC. In particular, the difficulty in bringing patients to the centre of the system was observed across all of the participants' settings. The study identified attitudinal and practical barriers to implementing patient‐centred approaches such as professionals recurring to paternalistic practice when confronted with a lack of resources, or the variability in patient willingness to be involved in decision making when they lack previous experience, skills or health literacy. These barriers had been previously identified in studies exploring perspectives regarding self‐care strategies of other populations such as health‐care professionals and patients.^5^, ^15^, ^16^, ^17^ The fact that the same sources of paternalistic attitudes and practices towards caring for chronic illness had been consistently identified as a deterrent for self‐care from a variety of perspectives reinforces the grounds for suggesting that actions directed at addressing latent causes of patient disempowerment could be sound and acceptable for enhancing SSSC and consequently for spreading the practice of self‐care.

Study findings also suggest that patient relegation to a minor role in the SSSC is also perpetuated by the lack of capacity, representativeness and independence of patient organizations for serving as the means for patients to influence health professionals' practices and health‐care policies. Concerns regarding the capacity of European patient organizations to advocate for patients have been raised before, but not in terms of its impact on the configuration of SSSCs.^18^ Thus, this study provides novel evidence to bring to light an important area for intervention if SSSC is to be redesigned as patient‐centred and to be truly supportive of self‐care strategies.

Another finding observed across the studied settings is the distortion in macro‐level stakeholders' practices and the relationships with stakeholders at the micro‐level. The inability of industry and the media to self‐regulate and reconcile their interests with society's health interests have been denounced previously and it appears to constitute an enduring problem requiring genuine attention from authorities.^19^, ^20^, ^21^

Thus, the cross‐country examination carried out in this study suggests that there are common enduring barriers to enacting changes in health systems which must be addressed to implement new strategies for tackling chronic illness, such as self‐care. Study key informants assigned the ultimate responsibility for addressing these barriers to governments and authorities, who should strive for truly enacting patient‐centred care by strengthening their strategies to address the latent factors behind the inclination of professionals and patients towards paternalistic and medicalized approaches to care; and the marginalization of patient capacity to influence health services and policies. In other words, authorities were said to be responsible for promoting industry and the media social responsibility, as well as patient associations' growth (so they can become more independent and focus on advocating for patients).

The traditional strategies of governments for encouraging corporate social responsibility, namely ministerial leadership in identifying and allocating risks, development of public–private partnerships, subsidy of corporate social responsibility activities and organizations, and development of soft regulation, have shown limited effectiveness.^22^ However, recent evaluations of public–private partnerships aimed at improving corporate social responsibility have provided valuable insight into what can be done to improve the strategies for engaging companies and producing environmental gains for public health. In particular, their effectiveness could be improved if they evolve, becoming evidence‐based, measurable and widespread, open to public and formal scrutiny, and supported by both appealing incentives (i.e. opportunities for improving organizational reputation) and sanctions for lack of commitment.^23^, ^24^

Moreover, to bolster the patient associations' independent growth and advocacy capacity, researchers and patient associations have often recommended that governments increase funding and facilitate access of patient associations to decision‐making structures. However, this governmental interference is not without disadvantages: patient associations may find it difficult to maintain patient agenda when facing the need for meeting criteria attached to subsidies; to oppose government proposals; or to use non‐institutional or activist oppositional strategies to influence decision making. For this reason, recent literature proposes that governments create less constrained subsidies that ‘come with fewer strings attached’, last longer periods of time and respond to public expenditure account systems that take into account a broad set of metrics that also evaluates their activities in terms of relevance for their members.^25^

In this research, the involvement of a range of countries with different socio‐economic, institutional and health‐care contexts posed major advantages for improving the trans‐European understanding of how SSSCs should be characterized and transformed. However, it also posed challenges for data collection and analysis that required attention so as to preserve the trustworthiness and dependability of the findings. Among them, the lack of equivalence of key concepts and the variability in the composition of the samples between the participating countries stood out. Attention to conceptual nuances was maintained in the adaptation of the interview guides and in the development of the common thematic framework and cross‐cultural data analysis clinics. The research team members had knowledge of the structural and cultural aspects of different countries so that interpretation errors stemming from cultural misunderstandings could be avoided. Moreover, efforts were made to minimize variability across samples that do not respond to national particularities that need to be accounted for in the development of intracountry relevant samples. For example, sampling biases associated with snowballing techniques for identifying potential key informants were prevented by complementing the sampling process with a review of policy statements and organizational websites in each partner country.

## Conclusion

Further development and spreading of new strategies for tackling chronic illness such as self‐care should be carried out if the chronic illness pandemic is to be contained and the sustainability of health systems is to be guaranteed. To achieve this, SSSCs should be reoriented to truly support a patient‐centred approach to caring that facilitates the effective involvement and coordination of multiple stakeholders at different levels. This requires a change in the persisting paternalistic and medicalized attitudes among patients and professionals, the growth and maturation of patient associations, and an increase in the social responsibility of both media and industry. European authorities play a critical role in creating environments that support public participation. They should develop and introduce enhanced public–private partnerships to improve industry and the media social responsibility and to offer less constrained subsidies and modes of participation for patient associations so they can be more capable of advocating for patients.

## Source of funding

This project has received funding from the European Union's Seventh Framework Programme for research, technological development and demonstration under grant agreement no 279081.

## Conflict of interest

No conflicts of interest have been declared.
